# Source Attribution of Human *Campylobacter* Isolates by MLST and *Fla*-Typing and Association of Genotypes with Quinolone Resistance

**DOI:** 10.1371/journal.pone.0081796

**Published:** 2013-11-14

**Authors:** Sonja Kittl, Gerald Heckel, Bożena M. Korczak, Peter Kuhnert

**Affiliations:** 1 Institute of Veterinary Bacteriology, Vetsuisse Faculty, University of Bern, Bern, Switzerland; 2 Institute of Ecology and Evolution, University of Bern, Bern, Switzerland; 3 Swiss Institute of Bioinformatics, Lausanne, Switzerland; Cornell University, United States of America

## Abstract

Campylobacteriosis is the most frequent zoonosis in developed countries and various domestic animals can function as reservoir for the main pathogens *Campylobacter jejuni* and *Campylobacter coli*. In the present study we compared population structures of 730 *C. jejuni* and *C. coli* from human cases, 610 chicken, 159 dog, 360 pig and 23 cattle isolates collected between 2001 and 2012 in Switzerland. All isolates had been typed with multi locus sequence typing (MLST) and *flaB*-typing and their genotypic resistance to quinolones was determined. We used complementary approaches by testing for differences between isolates from different hosts with the proportion similarity as well as the fixation index and by attributing the source of the human isolates with Bayesian assignment using the software STRUCTURE. Analyses were done with MLST and *flaB* data in parallel and both typing methods were tested for associations of genotypes with quinolone resistance. Results obtained with MLST and *flaB* data corresponded remarkably well, both indicating chickens as the main source for human infection for both *Campylobacter* species. Based on MLST, 70.9% of the human cases were attributed to chickens, 19.3% to cattle, 8.6% to dogs and 1.2% to pigs. Furthermore we found a host independent association between sequence type (ST) and quinolone resistance. The most notable were ST-45, all isolates of which were susceptible, while for ST-464 all were resistant.

## Introduction

Campylobacteriosis is the most frequent zoonosis in the European Union as well as in Switzerland [1]. The disease is characterized by fever, abdominal pain and diarrhea and is normally self-limiting [2]. However, *Campylobacter* infection might also be a prominent risk factor for Guillain-Barré syndrome and the development of inflammatory bowel disease, including Crohn’s disease, which highlights the importance of controlling this pathogen [2-4]. Campylobacteriosis is mainly caused by *C. jejuni*, which is responsible for about 91% of human cases in Switzerland, while the closely related species *C. coli* causes the remaining 9%, with other *Campylobacter* species being very rarely detected [5,6]. Both *C. jejuni* and *C. coli* live as commensals in various mammal and bird species which create many possible sources for human infection [7]. Isolates from wild birds were found to constitute a genetically distinct population compared to isolates from humans and domesticated animals, making wild birds an unlikely source of infection [8]. Most studies agree that the majority of human cases can be traced back to the chicken reservoir with variable contributions of other farm animals such as cattle, sheep and pigs, where the latter almost exclusively hosts *C. coli* [9]. Another potential reservoir, mainly for *C. jejuni*, is dogs, however, so far there are only few studies looking at this host [10,11]. In Switzerland there is a high prevalence of *Campylobacter* in pig herds (65%) and chicken flocks (33%-44%), while prevalence in cattle (15% in veal calves) and healthy dogs (6%) is much lower [6,11].

Among other methods, such as epidemiological approaches and expert elicitation, microbial subtyping can be used for source attribution of human cases [9,12]. Here, multi locus sequence typing (MLST) is the most widely applied method for *Campylobacter* as it is highly reproducible, can easily be compared between different studies and a public database is available at PubMLST [13]. Another genotyping method that is used less frequently with *Campylobacter* is *fla-*typing, which is based on the sequence of the short variable region (SVR) of the flagellin-encoding gene *flaA* or *flaB* and has a discriminatory index comparable to MLST [14,15]. The *fla* alleles are also publicly hosted and curated at the PubMLST database.

Due to the high diversity of *C. jejuni* and *C. coli* and the low host specificity of most genotypes, estimating the contribution of different sources to human infection is challenging. In addition, genotypes vary between countries of sampling and over time [16]. Several approaches have been applied to assess the similarity of *Campylobacter* isolates from different hosts, for example by using the proportion similarity index (PSI) [17,18] or the fixation index (F_ST_) [8]. Other approaches go one step further and provide population genetics estimates for the fractions of human cases attributable to each source e.g. based on the asymmetric island model [19] or Bayesian genotypic assignment with the software STRUCTURE [20,21].

Another important aspect, from a public health point of view, is the frequent antibiotic resistance of *Campylobacter* towards quinolones, which in most cases is due to the single mutation C257T in the *gyrA* gene [22]. This mutation carries apparently no fitness cost and therefore, resistance is quickly established following quinolone use in food animals [23]. This is reason for concern, as quinolones would also be considered for treatment in severe human infections [2]. 

Here we present a population genetic analysis of *C. jejuni* and *C. coli* isolates collected in Switzerland over a twelve year period from human cases and the potential sources chicken, dog, pig and cattle. We combine comprehensive data from their MLST alleles, the *flaB* SVR and genetic assessment of quinolone resistance which had been determined during previous studies [5,11,15,24-27]. Multiple analysis approaches were implemented to address the question of source attribution of human campylobacteriosis. First, as a simple measure to compare the overlap of sequence types (STs) between hosts, proportion similarity indices (PSIs) were calculated. Then, to take into account all the information present in the DNA sequences, fixation indices (F_ST_) between populations were determined. And finally, genotypic assignment with STRUCTURE [20] was applied to estimate the proportion of human cases attributable to each source. In addition, we compared the results obtained using either the MLST alleles or the *flaB* SVR. And finally we investigated whether quinolone resistance is associated with certain genotypes.

## Material and Methods

### Isolates

A total of 1243 *C. jejuni* and 616 *C. coli* isolates collected between 2002 and 2012 in Switzerland were included in the main analyses. For one *C. jejuni* and three *C. coli* isolates no unambiguous *flaB* sequence was available and they were excluded from analyses involving these sequences. The *C. jejuni* were isolated from dogs (159 isolates), chickens (435 isolates) and human campylobacteriosis cases (649 isolates), and have mostly been described previously [5,11,15,24,25,27]. The *C. coli* were isolated from pigs (360 isolates), chickens (175 isolates), and human clinical cases (81 isolates) and have also been characterized previously [5,15,25-27]. Briefly, chicken and pig isolates were collected at slaughterhouses in the framework of monitoring programs, while dog isolates stemmed from fecal samples submitted to our in-house diagnostics department, with the exception of 23 isolates from 2002/3 and 19 samples from 2012, which were taken from healthy dogs. Human case isolates were provided by the Swiss National Centre for Enteropathogenic Bacteria (NENT), in the framework of collaborative studies. 

For an additional source attribution analysis 23 available Swiss *C. jejuni* isolates taken from healthy dairy cows in 2001/2 were included [15]. This group was only used in the source assignement by MLST to get an idea if cattle are possibly important. They were not considered in the main analyses due to the bias of the small sample size and limited time span of only one year. A list of all included isolates is provided in the supplementary material (Table S1, Table S2).

Sampling has been approved by the Federal Office of Public Health for the human isolates and by the Federal Veterinary Office for the animal isolates. All patient data as well as data on farms and slaughterhouses where samples originated were anonymized.

### Statistical Analyses

The PSI as a measure for the overlap of two frequency distributions [28] was used to compare the MLST sequence types (ST) and *flaB* types of isolates from different sources. PSI ranges from zero to one, where one indicates that the two groups are identical and zero means they share no types. It was calculated according to: $PSI=1-0.5\sum_{1}^{i}|p_{i}-q_{i}|$where *p*_*i*_ and *q*_*i*_ are the proportion of isolates from group p and q, respectively, belonging to type i. Confidence intervals (CI) were estimated with the bootstrap method implemented in C++ as follows: The standard deviation (SD) of 1000 resampling rounds was calculated and the 95% CI estimated with PSI±2*SD. 

Analysis of molecular variance (AMOVA) and calculation of fixation indices (F_ST_) were performed with the Arlequin software [29]. Briefly, AMOVA partitions overall molecular variance into variance between individuals within populations, among populations within groups and among groups of populations. F_ST_ is a measure for genetic differentiation between populations ranging from zero to one where zero means that the populations are not separated. Both F_ST_ calculations and AMOVA were done using either *flaB* fragment sequences or concatenated sequences of the seven MLST alleles.

The STRUCTURE software [20] was used to assign the human isolates to their most probable source. Briefly, the most likely origin of the isolates is estimated based on the similarity in allele frequencies with the potential source populations. For the MLST data the program was run with 100,000 burn-in steps and 100,000 repeats applying the no-admixture model and assuming uncorrelated gene frequencies. The POPFLAG was set to zero for the human isolates and USEPOPINFO as well as STARTATPOPINFO turned on. This way, the program incorporated information on the population of origin for the isolates of the different potential sources, while the origin of the human isolates was presumed unknown. To test for the influence of single loci on the assignments, we ran jackknife analyses where each locus was left out once and the program was run with the remaining six loci as described above, except that burn-in steps and repeats were reduced to 10,000 to moderate run time. 

Bootstrap confidence intervals for source attribution were estimated using the following procedure implemented in Perl: STRUCTURE was run 100 times each time using a new subset of 100 human isolates randomly drawn with replacement from the whole set. For the resulting 100 assignment proportions, the mean and standard deviation were calculated and the 95% CI estimated with mean±2*SD. For the bootstraps, burn-in steps and repeats were also reduced to 10,000 to moderate run time, while the other parameters were left as before.

Self-attribution probabilities for the isolates from different animal hosts were calculated like the bootstrap confidence intervals for assignment of the human isolates, now taking a random sample of 100 draws with replacement of each isolate group and assigning them to a separate population. The POPFLAG for this test population was set to zero and the program was run as described above, calculating the probability of assignment to the correct source population.

As an attempt to assign the human isolates to their most probable source according to their *flaB* sequence, STRUCTURE was run using all polymorphic sites (153 in our dataset) in the *flaB* SVR. For this analysis, *C. jejuni* and *C. coli* were not separated because *flaB*-types are shared between both species, in contrast to STs, which are species-specific. However, to account for the larger variability of *flaB* over time (see results section) and avoid bias due to the unequal temporal distribution in the different sources, only isolates from 2008 to 2012 were included. Thereby, no cattle isolates were available for the *flaB* analyses. Parameters were set as described for the MLST data, except that burn-in steps and repeats were again reduced to 10,000.

Graphical displays of STRUCTURE results were created with the DISTRUCT software [30]. Derivation of binominal exact confidence intervals as well as standard logistic regression and penalized maximum likelihood logistic regression were performed with STATA IC 12.1 (StataCorp LP, Tx, USA).

## Results

### PSI

The proportion similarity indices (Table 1) for MLST types between *C. jejuni* from different sources were generally high with the greatest similarity between human and chicken isolates. The overlap between human and dog isolates was significantly lower, while dog and chicken isolates showed a high similarity. Comparable results were obtained for *flaB* types (Table 1). 

**Table 1:** Comparison of genotype frequencies between isolates from different hosts.

***C. jejuni***	**PSI_ST**	**PSI_*flaB***	F_ST_ **_MLST**	F_ST_ **_*flaB***
human-chicken	0.61 (0.55-0.66)	0.68 (0.63-0.73)	0.056 (0.049-0.063, p<0.001)	0.016 (0.012-0.019, p<0.001)
human-dog	0.46 (0.39-0.52)	0.56 (0.48-0.63)	0.082 (0.069-0.096, p<0.001)	0.020 (0.014-0.025, p<0.001)
chicken-dog	0.57 (0.50-0.64)	0.70 (0.63-0.77)	0.010 (0.008-0.013, p=0.02)	0.002 (0.001-0.003, p=0.18)
***C. coli***	**PSI_ST**	**PSI_*flaB***	F_ST_ **_MLST**	F_ST_ **_*flaB***
human-chicken	0.44 (0.35-0.53)	0.52 (0.43-0.61)	0.021 (0.007-0.041, p=0.001)	0.004 (0.002-0.006, p=0.26)
human-pig	0.11 (0.06-0.16)	0.15 (0.10-0.21)	0.167 (0.097-0.226, p<0.001)	0.239 (0.219-0.257, p<0.001)
chicken-pig	0.32 (0.25-0.38)	0.35 (0.28-0.41)	0.137 (0.083-0.183, p<0.001)	0.198 (0.180-0.216, p<0.001)

95% confidence intervals are indicated in brackets. For the fixation indices the p-value for the null-hypothesis of zero difference is also indicated. PSI=proportion similarity index, F_ST_ = fixation index

PSIs were generally lower between *C. coli* from different sources both for the MLSTs and *flaB* types (Table 1). Nevertheless, the overlap between human and chicken isolates was significantly higher than between human and pig isolates, while the similarity of chicken and pig isolates lay somewhere in between. 

In all comparisons the overlap of *flaB* types was higher than of MLSTs though never significantly so.

### F_ST_

Fixation indices were calculated between the same isolate groups as the PSIs (Table 1) using both the concatenated sequences of the seven MLST alleles and the *flaB* sequences alone. With this method *C. jejuni* isolates from humans were again determined to be closer to those from chickens than to those from dogs. Chicken and dog isolates were found to be very close with an F_ST_ of 0.010 (p=0.02) for the MLST sequences and an F_ST_ not significantly different from zero for the *flaB* sequences (p=0.18). In this instance the result differs from that obtained with the PSI, where the correspondence between human and chicken isolates was approximately the same as between dog and chicken isolates.

For *C. coli* human and chicken isolates were also found to be very closely related (Table 1). In contrast, there was a marked difference between human and pig isolates. As observed with the PSI, the difference between chicken and pig isolates was slightly lower. 

### Source assignment

**Figure 1:**
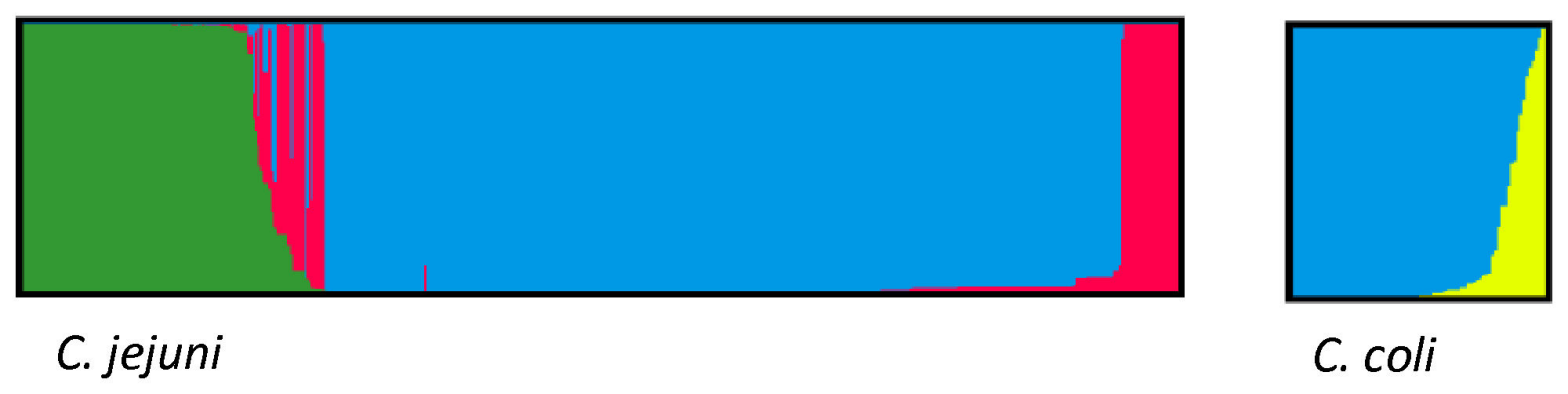
STRUCTURE source attribution of human *C. jejuni* and *C. coli* isolates. Single isolates are represented by vertical lines; the probability of attribution to the different source clusters is indicated by the color. Attribution to the chicken cluster is shown in blue, cattle cluster in green, dog cluster in red and pig cluster in yellow.

For the MLST data the STRUCTURE program was run separately for *C. jejuni* and *C. coli* because the human samples did not in all years represent an unbiased species distribution from all human cases; in 2008, for example, analyses focused on *C. jejuni* [24]. For *C. jejuni* 76.8% (95% CI: 64.7%-85.0%) of the human isolates were assigned to the chicken reservoir while 23.2% (95% CI: 15.0%-35.3%) were assigned to the dog reservoir. Our jackknife analyses showed that these STRUCTURE source attributions were robust to the exclusion of single alleles (Table S3). When this analysis was repeated including the 23 cattle isolates, 69.3% (95% CI: 57.5%-72.3%) of the human isolates were attributed to chicken, 9.5% (95 %CI: 8.8%-19.0%) to dogs and 21.2% (95% CI: 13.7%-28.7%) to cattle. For *C. coli* 86.4% (95% CI: 81.1%-92.3%) of the human isolates were estimated to stem from chickens and 13.6% (95% CI: 7.7%-18.9%) from pigs. Considering that 91% of human cases are caused by *C. jejuni* and 9% by *C. coli* [5], this suggests that in total 77.5% of the human campylobacteriosis cases can be attributed to the chicken reservoir, 21.1% to the dog reservoir and 1.4% to the pig reservoir, if cattle isolates are not considered. With the inclusion of the cattle isolates still 70.9% of the cases are attributed to chickens, but only 8.6% are attributed to dogs, 19.3% to cattle and 1.2% to pigs (Figure 1). 

Self-attribution was highest for *C. coli* in chickens with 79.8% (95% CI: 74.0%-85.6%) and slightly lower for *C. coli* in pigs with 70.1% (95% CI: 63.2%-77.0%). *C. jejuni* chicken isolates showed a self-attribution of 62.5% (95%CI: 53.7%-71.3%) while it was only 38.1% (95% CI: 30.3%-45.9%) in dog isolates and 26.1% (95% CI: 17.6%-34.5%) in cattle isolates. 

When STRUCTURE was run with the *flaB* sequences analyzing *C. jejuni* and *C. coli* together (since they share alleles), similar results were obtained. In this analysis 80.5% of the cases were assigned to the chicken reservoir, 17.7% to the dog reservoir and 1.8% to the pig reservoir. However, for *flaB* the separation between dog and chicken isolates was even less clear and dog isolates were mainly attributed to the chicken cluster.

### *Campylobacter* population turnover

To analyze the genotype variation over the years, AMOVA was performed with the concatenated MLST allele sequences as well as the *flaB* sequences (Table 2) for samples of 10 and more per year and host. For *C. jejuni* 4.6% of overall variance in MLST sequences was due to variance among hosts while only 1.6% was due to variance among years within hosts. The opposite was true for *flaB* sequences where only 0.6% of overall variance was due to variance among hosts and 2.0% was due to variance among years within hosts.

**Table 2:** Analysis of molecular variance (AMOVA) when grouping isolates according to host and year of isolation using either concatenated MLST sequences or *flaB* sequences.

	**assigned variance**	
***C. jejuni***	**MLST**	***flaB***
among years within hosts	1.6% (p<0.001)	2.0% (p<0.001)
among hosts	4.6% (p=0.008)	0.6% (p=0.178)
within hosts and years	93.8% (p<0.001)	97.4% (p<0.001)
***C. coli***		
among years within hosts	10.6% (p<0.001)	24.6% (p<0.001)
among hosts	7.9% (p=0.089)	7.4% (p=0.603)
within hosts and years	81.5% (p<0.001)	68.0% (p<0.001)

For each source only years with more than 10 samples were included.

In *C. coli* variance among years within hosts was much more pronounced, accounting for 10.6% of overall variance in MLST sequences and 24.6% in the *flaB* sequences (Table 2). The ST shift occurred somewhat in parallel between hosts. For example the frequency of ST-825 and ST-827 increased in both human and chicken isolates over the observed time period while there was a decrease in the frequency of ST-854 which was also observed in the pig isolates (Table S4).

### Comparison of quinolone resistant and susceptible isolates

AMOVA was performed for *C. jejuni* and *C. coli* separately to determine if MLSTs and *flaB*-types differ between quinolone resistant and susceptible strains (Table 3). In *C. jejuni* 5.9% of overall variance between the concatenated MLST allele sequences was due to differences between resistant and susceptible isolates within hosts, while there was no significant difference between hosts. Similar results were obtained for *flaB* sequences, where 8.0% of variance was due to variance between resistant and susceptible isolates within hosts. In *C. coli* the difference between quinolone susceptible and resistant isolates was smaller though it was still significantly different from zero (p <0.001; Table 3) while there was again no significant difference between hosts. When looking at the isolates from all hosts together, the F_ST_ for MLST between quinolone resistant and susceptible isolates of 0.067 (p<0.001; 95%CI: 0.058-0.076) suggests considerable differences in sequence types for *C. jejuni*, whereas this was significantly lower for *C. coli* (F_ST_ =0.017; p<0.001; 95%CI: 0.011-0.023).

**Table 3:** Analysis of molecular variance (AMOVA) when grouping isolates according to host and quinolone resistance using either concatenated MLST sequences or *flaB* sequences.

	**assigned variance**	
***C. jejuni***	**MLST**	***flaB***
between resistant and susceptible isolates within hosts	5.9% (p<0.001)	8.0% (p<0.001)
among hosts	1.9% (p=0.327)	-3.4% (p=0.802)
within hosts and resistance type	92.2% (p<0.001)	95.4% (p<0.001)
***C. coli***		
between resistant and susceptible isolates within hosts	3.6% (p<0.001)	3.2% (p<0.001)
among hosts	11.0% (p=0.133)	17.9% (p=0.134)
within hosts and resistance type	85.4% (p<0.001)	78.9% (p<0.001)

**Figure 2:**
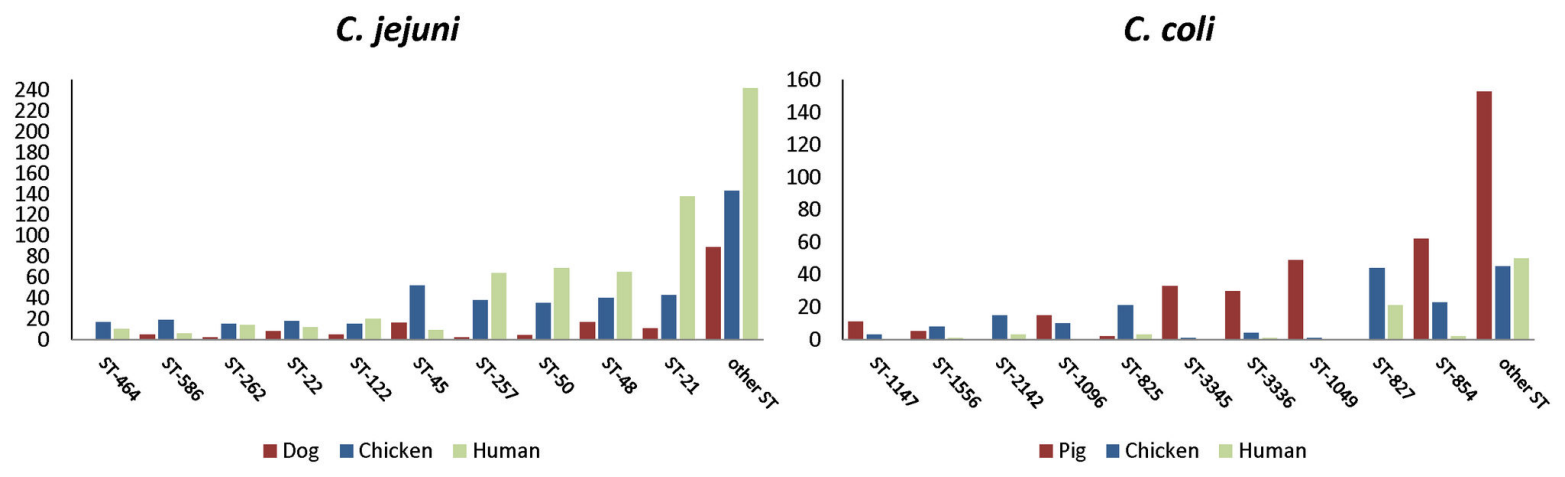
Frequency and origin of the ten most frequent *C. jejuni* and *C. coli* sequence types (ST) used in the regression analyses. The remaining 157 *C. jejuni* and 109 *C. coli* types were pooled in the category ‘other ST’. Numbers on the y-axis indicate the number of isolates.

To detect possible associations between ST and quinolone resistance in *C. jejuni* a logistic regression model was fitted including host and ST as risk factors (Table 4). The ten most frequent STs, together representing 61.9% of all isolates, were considered separately and compared to the remaining 157 pooled STs (Figure 2). Since all isolates with ST-45 (n=77) were quinolone susceptible and all ST-464 isolates (n=27) were resistant, the standard logistic regression model could not be applied. Instead, Firth’s penalized likelihood approach to separation was used to account for the problem of separation [31]. Isolates from humans had significantly higher odds of being resistant, than those from chickens or dogs. Eight of the ten most frequent STs were associated with significantly lower quinolone resistance, with the lowest OR for ST-45. ST-464 was associated with significantly higher odds of quinolone resistance. 

**Table 4:** Logistic regression for quinolone resistance in *C. jejuni*.

**host**	**OR**	**p-value**	**95% CI**
dog	0.956	=0.857	0.589-1.554
human	2.448	<0.001	1.782-3.364
**ST**			
21	0.824	=0.283	0.58-1.172
22	0.104	=0.001	0.028-0.385
45	0.012	=0.002	0.001-0.198
48	0.169	<0.001	0.094-0.303
50	0.974	=0.903	0.631-1.501
122	0.120	<0.001	0.039-0.368
257	0.159	<0.001	0.085-0.297
262	0.113	=0.001	0.03-0.422
464	93.55	=0.002	5.623-1556
586	0.087	=0.004	0.017-0.458

Chicken was specified as baseline host. The ten most frequent sequence types were compared to the pooled rest as baseline. OR = odds ratio, CI = confidence interval for OR

For *C. coli* the ten most frequent STs, accounting together for 59.7% of all isolates (Figure 2), were also used to fit a logistic regression model for quinolone resistance but here the standard model could be applied. Chicken was specified as the baseline host and the remaining pooled STs (109 different types) were used as baseline (Table 5). Again human isolates had significantly higher odds of resistance than chicken isolates but also pig isolates had higher odds. Five of the ten most common STs were associated with significantly lower odds of resistance. 

**Table 5:** Logistic regression for quinolone resistance in *C. coli*.

**host**	**OR**	**p-value**	**95%CI**
pig	1.687	=0.032	1.044-2.724
human	4.328	<0.001	2.355-7.954
**ST**			
825	0.635	=0.343	0.249-1.622
827	0.272	<0.001	0.131-0.563
854	0.847	=0.522	0.510-1.408
1049	0.069	<0.001	0.021-0.231
1096	2.375	=0.052	0.994-5.677
1147	0.091	=0.022	0.012-0.709
1556	1.839	=0.288	0.598-5.653
2142	0.257	=0.046	0.067-0.978
3336	0.273	=0.004	0.114-0.656
3345	0.521	=0.100	0.239-1.133

Chicken was specified as the baseline host. The ten most frequent sequence types were compared to the pooled rest as baseline. ST = sequence type OR = odds ratio, CI confidence interval for OR

A temporal analysis showed a tendency towards increasing quinolone resistance over time for dog and chicken isolates, while there was little change among human and pig isolates (Table S5).

## Discussion

We applied complementary approaches based on two genotyping methods to determine the most likely source for human *Campylobacter* infection in Switzerland. The accordance between methods and approaches was high, as all indicated the closest genetic relationship of *C. jejuni* and *C. coli* strains from humans with chicken isolates. In addition, the STRUCTURE software assigned more than 70% of human cases to the chicken reservoir. A large contribution of the chicken reservoir is also plausible in light of the high prevalence (>50%) of *Campylobacter* on Swiss chicken retail meat [32]. The proportion attributed to chicken is comparable to findings in other countries: Mullner et al. [33], for example, estimated poultry to be the source of 76% of human cases in New Zealand, Sheppard et al. [21] attributed up to 78% of human cases in Scotland to chickens and Wilson et al. [19] attributed 56.5% of cases in the UK to chickens. However, none of these studies included dogs as a possible source. A recent study from The Netherlands, which did include pets (dogs and cats), obtained similar results to ours with 63% attribution to chickens, 25% to pets, 11% to cattle, 0.5% to pigs, 0.4% to sheep and 0.1% to the environment [10]. In our study between 21.1% and 8.6% of the cases were attributed to dogs. The attribution to the cattle reservoir (19.3%) of our samples was also comparable to findings in other countries (18%, 18-38% and 35%, respectively) [19,21,33]. However, this result has to be viewed with caution due to the small sample size and limited time span represented by the cattle isolates. In Switzerland *Campylobacter* prevalence in cattle is comparatively low (15% in veal calves in 2010 as opposed to e.g. 65% in pigs ) and analogous to the pig reservoir, infection would have to result from direct contact since veal, beef and pork are very rarely contaminated with *Campylobacter* [1]. Raw milk, which might also harbor *Campylobacter*, is only rarely consumed in Switzerland [32]. Infection through drinking water, which has been observed in other countries [34], is unlikely to play a major role in Switzerland as all drinking water originating from surface waters is treated and spring, as well as ground water were found to contain no *Campylobacter* [32]. 

The very poor self-attribution of dog isolates might indicate that chickens are an important source of *Campylobacter* for dogs as well. Thus, the indication of dogs as a reservoir for human infection could be misleading and finally be due to a common chicken reservoir, especially, since the prevalence in healthy dogs is comparatively low with 6% [11]. That these two isolate populations from chickens and dogs are very close is also indicated by the F_ST_ for *flaB*, which is not significantly different from zero. Nevertheless, the two populations do not seem to be completely overlapping since the F_ST_ for MLST was significantly different from zero.

The case is much clearer for the pig reservoir, which plays a very minor role in human infection. Only 1.4% of the human cases were attributed to pigs with MLST and 1.8% with *flaB*. Studies in other countries came to similar conclusions: Wilson et al. [19] estimated the pig reservoir to be responsible for 0.8% of human cases and Sheppard et al. [21] also found only little contribution of the pig reservoir to human infection. 

We could not exclude potentially travel associated cases, since case histories were only available for isolates from 2009. This might also slightly bias source attribution because the ST distributions can differ between countries [16]. In Switzerland, approximately 18% of the cases were travel associated in 2009 [5]. 

Variation over years was much more pronounced in *C. coli* than in *C. jejuni* and there were parallels between the different hosts. It remains unclear why this shift in genotypes occurred; it illustrates, however, that comparisons with temporally distant isolates may bias source attribution. *flaB* genotype frequencies proved to be less stable over time as indicated by the higher variation assigned to year of isolation in AMOVA. For this reason we only used isolates from a four year period (2008-2012) for the STRUCTURE assignment with *flaB*. Thus, MLST would probably be the preferred method for long term studies, especially, as it has been shown that MLST is well correlated with the core genome [35].

Another interesting finding was the clear association between certain STs and quinolone resistance. This correlation was independent from the host, which suggests that some types, like ST-464, of which all isolates were resistant, represent strains that are intrinsically more likely to develop quinolone resistance. In *C. jejuni* the lowest odds ratio was observed for ST-45 where all isolates were susceptible. This confirms our previous observation with a subset of the here included isolates from chicken in 2008 [25]. Habib et al. [36] also found clonal complex 45, to which ST-45 belongs, to be associated with quinolone susceptibility, while isolates of clonal complex 21 were associated with resistance. In our study isolates with ST-21 and ST-50, which both belong to clonal complex 21, also had significantly higher odds of resistance than ST-45. Strains of ST-45 were hypothesized to be more environmentally adapted [37] and thus might come less frequently into contact with antibiotics, which might provide an explanation for their low quinolone resistance [36]. However, since ST-45 isolates are frequent in both chickens and dogs, they would also come into contact with quinolones, and if quinolone resistance is not associated with a loss of fitness in *Campylobacter* [23], one would expect it to rise in frequency over time. Another possible explanation is the low frequency of ST-45 among the Swiss human isolates [5,24], while humans are the host with the highest rate of quinolone resistant strains. This would, however, imply that a substantial proportion of strains would gain their resistance in the human host and then return to the chicken or dog reservoir, which seems an unlikely scenario. Moreover, in contrast to Switzerland, ST-45 is not rare among humans in other countries [16]. Therefore, a conclusive explanation for the absence of quinolone resistance in ST-45 must await further research.

Interestingly, in both *C. jejuni* and *C. coli* the odds of quinolone resistance were highest in isolates from humans. This might in part be due to travel associated infections from countries with higher resistance rates [5] or reflect antibiotic administration prior to sample submission. It is also possible that quinolone resistant strains are more pathogenic for humans. There is some evidence that quinolone resistant *Campylobacter* can lead to more severe disease but it is, however, unclear if this is due to treatment failure or different virulence characteristics of the strains [38]. 

In conclusion, we present the first study (to our knowledge) comparing in parallel the use of MLST allele and *flaB* SVR sequences for the source attribution of human isolates. Both methods were found to be valid and showed a remarkable congruence but *flaB* typing must be used with caution when comparing temporally distant isolates. We found that in Switzerland, as in many other countries, chickens can be considered the main source for human campylobacteriosis and efforts to reduce prevalence should focus on this reservoir. Our evidence of an association between ST and quinolone resistance suggests the need for more research in this area to clarify the underlying mechanisms and its implications from a public health point of view.

## Supporting Information

Table S1 **Isolates used in the main analysis by year and source.**
(DOCX)

Table S2 ***Campylobacter jejuni* and *Campylobacter coli* strains included in the study.** Individual information on species, host, genotype year of isolation is given.
(XLSX)

Table S3 **Proportions of human isolates assigned to the respective source populations by the STRUCTURE model when one locus was left out.**
(DOCX)

Table S4 **Temporal change in the frequency of *C. coli* ST-825, ST-827 and ST-854 in the three hosts.**
(DOCX)

Table S5 **Percentage of quinolone resistant strains by source and year.**
(DOCX)
