# Supplementary material for: Source Attribution of Human Campylobacter Isolates by MLST and Fla-Typing and Association of Genotypes with Quinolone Resistance
Source: PLoS One. 2013 Nov 14;8(11):e81796. doi: 10.1371/journal.pone.0081796 (PMC3828285; doi:10.1371/journal.pone.0081796)
Supplement: Table S1 — Isolates used in the main analysis by year and source. (DOCX) [file pone.0081796.s001.docx]

Table S1 Isolates used in the main analysis by year and source

| ***C. jejuni*** | | | | |
| --- | --- | --- | --- | --- |
|  | **source** | | | |
| **year** | **human** | **dog** | **chicken** | **total** |
| 2002 | 0 | 16 | 45 | 61 |
| 2003 | 0 | 15 | 2 | 17 |
| 2004 | 24 | 7 | 0 | 31 |
| 2005 | 4 | 1 | 0 | 5 |
| 2006 | 61 | 4 | 0 | 65 |
| 2007 | 0 | 8 | 0 | 8 |
| 2008 | 136 | 11 | 243 | 390 |
| 2009 | 414 | 25 | 145 | 584 |
| 2010 | 10 | 22 | 0 | 32 |
| 2011 | 0 | 13 | 0 | 13 |
| 2012 | 0 | 37 | 0 | 37 |
| **total** | **649** | **159** | **435** | **1243** |
| ***C. coli*** | | | | |
|  | **source** | | | |
| **year** | **human** | **pig** | **chicken** | **total** |
| 2002 | 0 | 103 | 25 | 128 |
| 2004 | 17 | 0 | 0 | 17 |
| 2005 | 19 | 0 | 0 | 19 |
| 2008 | 3 | 0 | 98 | 101 |
| 2009 | 42 | 257 | 52 | 351 |
| **total** | **81** | **360** | **175** | **616** |
