# Supplementary material for: Source Attribution of Human Campylobacter Isolates by MLST and Fla-Typing and Association of Genotypes with Quinolone Resistance
Source: PLoS One. 2013 Nov 14;8(11):e81796. doi: 10.1371/journal.pone.0081796 (PMC3828285; doi:10.1371/journal.pone.0081796)
Supplement: Table S3 — Proportions of human isolates assigned to the respective source populations by the STRUCTURE model when one locus was left out. (DOCX) [file pone.0081796.s003.docx]

Table S3 Proportions of human isolates assigned to the respective source populations by the STRUCTURE model when one locus was left out

|  | **Locus**  **left out** | | | | | | | |
| --- | --- | --- | --- | --- | --- | --- | --- | --- |
| ***C. jejuni*** | ***tkt*** | ***gltA*** | ***aspA*** | ***atpA*** | ***glmM*** | ***glyA*** | ***glnA*** | **none** |
| assigned to chickens | 74.4% | 77.1% | 65.8% | 70.5% | 73.7% | 70.6% | 80.4% | 76.8% |
| assigned to dogs | 25.6% | 22.9% | 34.2% | 29.5% | 26.3% | 29.4% | 19.6% | 23.2% |
| ***C. coli*** |  | | | | | | | |
| assigned to chickens | 84.5% | 85.0% | 84.9% | 85.0% | 84.7% | 85.8% | 86.2% | 86.4% |
| assigned to pigs | 15.5% | 15.0% | 15.1% | 15.0% | 15.3% | 14.2% | 13.8% | 13.6% |
