# Supplementary material for: Source Attribution of Human Campylobacter Isolates by MLST and Fla-Typing and Association of Genotypes with Quinolone Resistance
Source: PLoS One. 2013 Nov 14;8(11):e81796. doi: 10.1371/journal.pone.0081796 (PMC3828285; doi:10.1371/journal.pone.0081796)
Supplement: Table S4 — Temporal change in the frequency of C. coli ST-825, ST-827 and ST-854 in the three hosts. (DOCX) [file pone.0081796.s004.docx]

Table S4 Temporal change in the frequency of *C. coli* ST-825, ST-827 and ST-854 in the three hosts.

| **ST825** | | | |
| --- | --- | --- | --- |
| **year** | **human** | **chicken** | **pig** |
| 2002 | - | 4% | 2% |
| 2004 | 0% | - | - |
| 2005 | 0% | - | - |
| 2008 | - | 12% | - |
| 2009 | 7% | 15% | 0% |
| **ST827** | | | |
| **year** | **human** | **chicken** | **pig** |
| 2002 | - | 0% | 0% |
| 2004 | 6% | - | - |
| 2005 | 11% | - | - |
| 2008 | - | 33% | - |
| 2009 | 43% | 23% | 0% |
| **ST854** | | | |
| **year** | **human** | **chicken** | **pig** |
| 2002 | - | 36% | 37% |
| 2004 | 6% | - | - |
| 2005 | 0% | - | - |
| 2008 | - | 9% | - |
| 2009 | 2% | 10% | 9% |
