# Supplementary material for: Source Attribution of Human Campylobacter Isolates by MLST and Fla-Typing and Association of Genotypes with Quinolone Resistance
Source: PLoS One. 2013 Nov 14;8(11):e81796. doi: 10.1371/journal.pone.0081796 (PMC3828285; doi:10.1371/journal.pone.0081796)
Supplement: Table S5 — Percentage of quinolone resistant strains by source and year. (DOCX) [file pone.0081796.s005.docx]

Table S5 Percentage of quinolone resistant strains by source and year

| ***C.jejuni*** | | | |
| --- | --- | --- | --- |
| **year** | **human** | **chicken** | **dog** |
| 2002 | - | 8.9% (2.5%-21.2%) | 0.0% (0%-20.6%) |
| 2003 | - | - | 20.0% (4.3%-48.1%) |
| 2004 | 45.8% (25.6%-67.2%) | - | - |
| 2005 | - | - | - |
| 2006 | 34.4% (22.7%-47.7%) | - | - |
| 2007 | - | - | - |
| 2008 | 37.5% (29.3%-46.2%) | 18.9% (14.2%-24.4%) | 18.2% (2.3%-51.8%) |
| 2009 | 42.3% (37.5%-47.2%) | 29.0% (21.7%-37.1%) | 12.0% (2.5%-31.2%) |
| 2010 | 40.0% (12.2%-73.8%) | - | 22.3% (7.8%-45.4%) |
| 2011 | - | - | 30.8% (9.1%-61.4%) |
| 2012 | - | - | 32.4% (18.0%-49.8%) |
| ***C.coli*** | | | |
| **year** | **human** | **chicken** | **pig** |
| 2002 | - | 0% (0%-13.7%) | 47.6% (37.6%-57.6%) |
| 2004 | 82.4% (56.6%-96.2%) | - | - |
| 2005 | 78.9% (54.4%-93.9%) | - | - |
| 2008 | - | 26.5% (18.1%-36.4%) | - |
| 2009 | 45.2% (29.8%-61.3%) | 40.4% (27.0%-55.0%) | 33.9% (28.1%-40.0%) |

95% confidence intervals (binominal exact) are indicated in brackets. Only years with at least 10 samples were included. ‘-‘ = not sampled/not included
